# Intensive Care and Emergency Nurses’ Attitudes and Stigma Towards Suicidal Behavior and Related Factors

**DOI:** 10.1192/j.eurpsy.2025.909

**Published:** 2025-08-26

**Authors:** N. Karabulut, Ç. Yüksel

**Affiliations:** 1 Department of Psychiatric Nursing, Ankara University; 2 Department of Psychiatric Nursing, Health Sciences University, Ankara, Türkiye

## Abstract

**Introduction:**

The first place to intervene in individuals who have attempted suicide is often the emergency department. These individuals may then be referred to intensive care units for close monitoring of physical findings and treatment, depending on the results of the suicide attempt. The attitudes and stigmatisation of nurses in the emergency department and intensive care unit toward suicide affect the quality of care provided. It is important to determine the attitudes and stigmatisation of nurses.

**Objectives:**

This study aims to determine the attitudes and stigma levels of nurses working in intensive care and emergency services towards suicidal behaviour and to examine the related factors.

**Methods:**

The research is descriptive, cross-sectional and correlational. The research was conducted with 82 volunteer nurses working in the emergency room and intensive care unit of a hospital in Turkey between January and March 2023, with approval from the ethics committee and the institution. Nurses filled out the Personal Information Form, Attitude Towards Suicide Attempt Scale and Stigma of Suicide Scale. In data analysis; Independent samples t-test, One-way ANOVA, and Welch test were used in cases where variances were not homogeneously distributed. Bonferroni and Games-Howell methods were preferred among post hoc multiple comparison tests. Pearson correlation was used for the relationship between continuous variables, and Spearman correlation analysis was used for categorical variables.

**Results:**

The mean score of the nurses’ Attitudes Towards Suicide Attempt Scale was 98.78±9.16. The mean scores of the Stigma of Suicide Scale; Stigma, Isolation\Depression, Sublimation\Normalization sub-dimensions were 71.95±18.09, 57.68±10.94, 27.11±6.86, respectively. The mean score of the Sublimation\Normalization sub-dimension of the Stigma of Suicide Scale of those who had attempted suicide in their close circle was higher than those who had not, and the results were significant (p<0.05). In the same sub-dimension, the mean scores of those who gave care to patients who attempted suicide were lower than those who did not, and the results were significant (p<0.05). The total score of the Attitude Towards Suicide Attempt Scale had a positive and statistically significant effect on the Stigma of Suicide Scale (β=0.562, t=6.071, p<0.001).

**Conclusions:**

These results indicate that nurses have high levels of negative attitudes towards suicidal behaviour, stigmatize it, associate suicide with depression and isolation, consider suicide normal, or glorify people who commit suicide. The presence of those who have attempted suicide in the immediate environment and providing care to individuals who have attempted suicide was associated with nurses’ attitudes and stigmatisation towards suicidal behaviour. It was found that nurses’ negative attitudes towards suicide had an increasing effect on their stigmatisation.

**Disclosure of Interest:**

None Declared

